# Supplementary material for: Signaling Cross-Talk between Salicylic and Gentisic Acid in the ‘Candidatus Phytoplasma Solani’ Interaction with Sangiovese Vines
Source: Plants (Basel). 2023 Jul 19;12(14):2695. doi: 10.3390/plants12142695 (PMC10383235; doi:10.3390/plants12142695)
Supplement: Supplementary file 1 [file plants-12-02695-s001.zip › Figure S1.pdf]

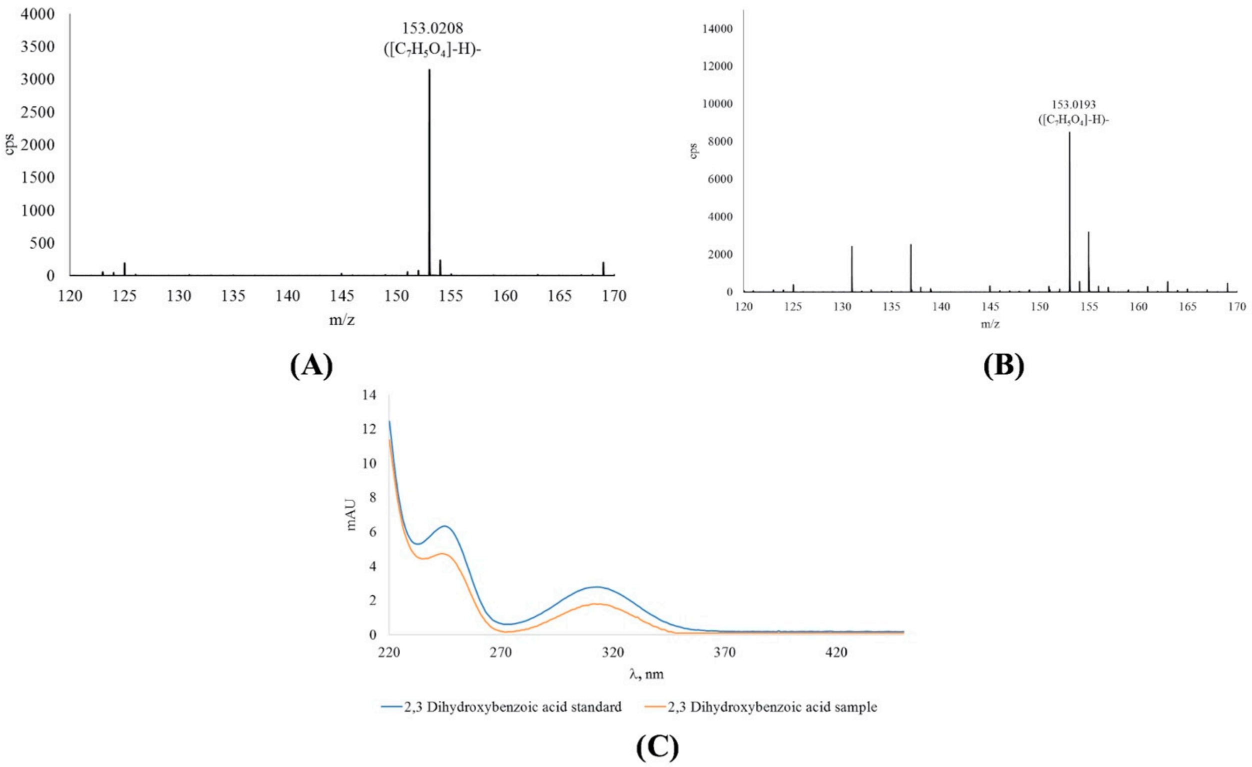

Figure S1. Mass spectra of the 2,3 Dihydroxybenzoic acid (2,3 DHBA) found in the sample (A) and of the chemical standard (B) and their UV/Vis absorption spectrum of the peak 8B shown in Fig. 1 after sample digestion with β-glucosidase (C).
