# Supplementary material for: Signaling Cross-Talk between Salicylic and Gentisic Acid in the ‘Candidatus Phytoplasma Solani’ Interaction with Sangiovese Vines
Source: Plants (Basel). 2023 Jul 19;12(14):2695. doi: 10.3390/plants12142695 (PMC10383235; doi:10.3390/plants12142695)
Supplement: Supplementary file 1 [file plants-12-02695-s001.zip › Table S1.pdf]

Table S1. Primer selected for the gene expression analysis.

Gene	Accession number	Sequence 5′-3′	Amplicon (bp)
PAL	XM_034831159.1	For: CAGAGCGCTGAGCAACACAA<br>Rev: TTTTCGCGAAGAGATCAATCC	64
C4H	XM_002266202.3	For: GGCAAGCACAAAGAGCACAGAT<br>Rev: TTCTTCTGGATGTGAGGGTGGTT	60
CHS1	X75969	For: GTGAAGCAGGTAGCCAACAATG<br>Rev: CTCAGCTCTCTGGGCCTTTCT	68
CHS2	XM_019224647	For: TCTAGTGCATGCGTGCTGTTT<br>Rev: TTTCCTTCTTCGATCGACTTTTTC	59
CHS3	XM_034830349	For: TCACTTGGACAGCCTTG<br>Rev: CAATTCGAACATGGGCTTCT	58
F3H1	XM_034827549	For: CCAATCATAGCAGACTGTCC<br>Rev: TCAGAGGATACACGGTTGCC	57
F3H2	XM_034820847	For: CGATTGCCACGTTCCAGAA<br>Rev: TCTCTGATCTTGAGTGG	57
FLS	XM_002285803	For: CATTCATGTCGGTGACCAGTTG<br>Rev: CTGTGAAGCACCGCCTTGT	60
F3′H	KT216254	For: TGGACCTGCCCCAACTCA<br>Rev: GGTGCAGGCGGAAGGTT	61
DFR	NM_001281215	For: CCTGTAGATGGCAAGACCTAGAAGA<br>Rev: TGATTCCACAGAATGAAGGACATT	60
LDOX	XM_034817321	For: GGAAGGGAAAACAAGTAGATCAGTGA<br>Rev: TGCTGGACAGGGCTCTCAACTCTAG	59
UF3GT	XM_034854803	For: ACAGGGCGGCCAGTGA<br>Rev: CCATATCTGCGGCAAACCAT	57
LAR	XM_034825191	For: GGAAAAGAAGATTGGGAGGACACT<br>Rev: CCGGCTGCAGCTAGTAGATCAT	60
NPR1	XM_002281439	For: TGATATCACGCCAGACGGTAGA<br>Rev: CAGCCCTGGTGAGCCTCTT	59
PR1	NM_001405846	For: GGAGCAAATCAGTGCGTCTTG<br>Rev: CGAACCACCCTCCATTGTTG	59
PR2	XM_034837447	For: TGCTTGTTTTTGGGCTGCTA<br>Rev: CTCCCGTTTGGGCAACTG	57
PR5	AF003007	For: ACGGTTCTCCCCCAAACAC<br>Rev: CGAGGTTATTGGGCTGGTTTAG	53
ICS	XM_034817670	For: TCTTCCCCGCTGTTTCTTCT<br>Rev: CTAAACCGTTGCCATCTCCG	89
DMR6	XM_034854199	For: TGGGGTAGCTGCAGAAATGA<br>Rev: ACCGGCAGTCTGTAGAACTC	66
COX	XM_003633668	For: AGTTGGGATTCGTCGTTTCTTC<br>Rev: TCTTTTGTTGTTTCCACTGCTTGA	59
